# Secreted clusterin (sCLU) regulates cell proliferation and chemosensitivity to cisplatin by modulating ERK1/2 signals in human osteosarcoma cells

**DOI:** 10.1186/1477-7819-12-255

**Published:** 2014-08-09

**Authors:** Hai Huang, Linna Wang, Mingyu Li, Xiaohui Wang, Lin Zhang

**Affiliations:** Department of Orthopedics, the Linyi People’s Hospital, Linyi, Shangdong 276003 China; Department of Clinical Laboratory, Qingdao Sanatorium of Shandong Province, Qingdao, China; Department of NICU, the Affiliated Hospital of QingDao University, QingDao, 266003 China; Department of Orthopedics, the Central Hospital of Yishui, Linyi, Shan Dong Province 276400 China; Department of Anesthesiology, the Affiliated Hospital of QingDao University, QinDao, 266003 China

**Keywords:** Osteosarcoma, Chemotherapy, Secreted clusterin, RNA interference, ERK activator kinase 1/2

## Abstract

**Background:**

Several studies have shown that secreted clusterin (sCLU) up-regulation in multi-drug resistant osteosarcoma (OS) cells relates to enhanced drug resistance. Furthermore, sCLU silencing directed against sCLU induces significant reduction of cellular growth and sensitizes OS cells to chemotherapy. However, the molecular mechanisms underlying the effect of sCLU on OS cells are not known.

**Methods:**

To evaluate the roles and possible mechanisms of sCLU in chemoresistance of OS cells to cisplatin (DPP), we utilized RNA interference to knockdown sCLU expression in the sCLU-rich U-2 OS cells and to overexpress sCLU in the sCLU-poorer KH OS cells, and further assessed the cell viability and chemosensitivity to DDP as well as possible signaling transduction pathways.

**Results:**

The data showed that sCLU depletion inhibited growth and sensitized sCLU-rich U-2 OS cells to cisplatin *in vitro* and *in vivo* by inducing inactivation of ERK1/2, and sCLU overexpression promoted growth and increased resistance of sCLU-less KH OS cells to cisplatin *in vitro* and *in vivo* by activation of ERK1/2.

**Conclusions:**

The data also suggests critical roles of sCLU in OS cell chemoresistance to DPP and raises the possibility of sCLU depletion as a promising approach to OS therapy.

## Background

Clusterin (CLU) is a ubiquitously expressed glycoprotein that has been implicated in a variety of physiological processes, including cell-cell interaction, lipid transport, tissue remodeling, chaperone activity, and apoptosis [1, 2]. CLU appears to have two main isoforms, which are the result of alternative splicing [3–5]. The secreted form of CLU (sCLU) starts as an approximately 60 kDa precursor peptide [6]. It is then cleaved and glycosylated to form an α and a β chain, each about 40 kDa, which ultimately become the heterodimeric secreted form [1]. sCLU is thought to be cytoprotective and may be involved with the clearance of cellular debris and promotion of phagocytosis [7, 8]. A nuclear form that is 49 kDa in its inactive form can be posttranslationally modified to an active 55 kDa form that accumulates in the nucleus and induces cell death [5, 9–12].

Studies have demonstrated that sCLU is pro-survival and plays a role in conferring chemoresistance in cancer cells [13–15]. It has recently been reported that sCLU knockdown in human cancer cells induces significant reduction of cellular growth and higher rates of spontaneous endogenous apoptosis. Moreover, sCLU silencing in cancer cells significantly sensitizes them to both genotoxic and oxidative stress induced by chemotherapeutic drugs and H_2_O_2_, respectively [16]. In three human osteosarcoma (OS) cell lines, namely, Sa OS, KH OS, and U-2 OS cells, sCLU silencing resulted in increased sensitization to deep radiation therapy (DXR)-induced apoptosis. Supportively, moderate levels of forced transgene-mediated CLU stable overexpression in KH OS cells could rescue them from DXR-mediated apoptosis. In contrast, stable overexpression of high CLU levels in Sa OS and U-2 OS cells augmented apoptosis induced by cell exposure to severe DXR-mediated genotoxic stress [17]. Lourda *et al*. also found sCLU up-regulation in the multi-drug resistant OS cells is related to enhanced drug resistance, and sCLU silencing could reverse the chemosensitivity [18]. However, the molecular mechanisms underlying the effect of sCLU on OS cell chemosensitivity is still unknown.

Extracellular signal-regulated protein kinases 1 and 2 (ERK1/2) are members of the mitogen-activated protein kinase super family that can mediate cell proliferation and apoptosis [19]. Furthermore, RNAi-mediated down-regulation of ERK1/2 expression can lead to potent antitumor activity and chemosensitizing effects in human pancreatic cancer and OS [15, 19]. Recent studies have demonstrated that ERK1/2 is regulated by CLU in renal cell cancer [20], pancreatic cancer [15], monocytes/macrophages [21] and lung cancer [22].

In the present study, we aimed to determine that the vector expressing short hairpin RNA against sCLU RNA (sCLU-shRNA) inhibits growth and enhances the chemosensitivity to DDP in sCLU-rich U-2 OS cells, and the vector expressing sCLU (pCDNA3.1-sCLU) promotes growth and increases chemoresistance to DDP in the lower sCLU-level KH OS cells. sCLU-mediated regulation of ERK1/2 activity leads to potent antitumor/pro-tumor activity and chemosensitizing/chemoresistant effects in human OS.

## Methods

### Cell lines and culture conditions

OS cell lines (KH OS, Sa OS and U-2 OS) were purchased from the American Tissue Culture Collection (ATCC). The cells were cultured in DMEM (Life Technologies, Inc., San Diego, CA, USA) supplemented with 10% fetal calf serum (FCS), 2 mM glutamine, and 1% nonessential amino acids (complete medium).

### Materials

Anti-ERK1/2 (Ab-202/204) and Anti-ERK1/2 (Phospho-Thr202/Tyr204) and anti-β-actin were purchased from Santa Cruz Biotechnology, Santa Cruz, CA, USA. Clusterin (A-9) (sc-166907, 1:200) was purchased from Santa Cruz (Shanghai, China). Horseradish peroxidase-conjugated goat anti-mouse and donkey anti-rabbit antibodies were obtained from Amersham Biosciences. DMEM/F12, RPMI 1640, FBS, and Normocin antibiotic were all purchased from InvivoGen. PD98059, a specific inhibitor of ERK kinase was purchased from Biovision, Beijing, China. Mitogen activated kinase 1 human recombinant (MEK1), the ERK activator kinase 1/2(ERK1/2), was from Shanghai, China. Cisplatin (DDP) was purchased from Zibo.co, Shandong, China.

### sCLU short hairpin RNA stable clones

Vector expressing short hairpin RNA against sCLU RNA (sCLU-shRNA1/2) plasmid and scrambled plasmid was purchased from Santa Cruz (Shanghai, China). When 50% to 60% confluence was reached, U-2 OS cells were transfected with the plasmids using the Lipofectamine 2000 (InvivoGen, Shanghai China) according to the manufacturer’s protocol. The stable clones (U-2 OS/sCLU-shRNA-1, U-2 OS/sCLU-shRNA-2 and control U-2 OS/shRNA) were selected by culturing transfected cells in the presence of 1.0 mg/mL G418 (InvivoGen, Shanghai, China). The knockdown effect was verified by Western blotting analysis using anti-sCLU antibody.

### Full-length human clusterin cDNA stable clones

Reverse transcription-PCR of normal human fibroblast total RNA was performed using the primers 5′-GACTCCAGAATTGGAGGCATG-3′ (forward) and 5′-ATCTCACTCCTCCCGGTGCT-3′ (reverse). The cDNA was cloned into pCRTMII (Invitrogen). CLU full-length cDNA was then subcloned into the pCDNA3.1 to produce the pCDNA3.1-sCLU vector. Constructs have been sequenced before carrying out expression experiments. When 60 to 70% confluence was reached, KH OS cells were transfected with the pCDNA3.1-sCLU plasmids using Lipofectamine 2000 according to the manufacturer’s protocol. The stable clones (KH OS/sCLU and control scrambled KH OS/pCDNA3.1) were selected with 400 μg/mL G418 for 14 days. Selected colonies were screened by immunoblotting to identify stable clones expressing sCLU.

### Drug treatments

KH OS, Sa OS, U-2 OS, KH OS/pCDNA3.1-sCLU, KH OS/pCDNA3.1, U-2 OS/sCLU-shRNA1/2 and U-2 OS/shRNA cells were treated with cisplatin (1 to 10 μM/mL) for 72 hours or with the same concentration of cisplatin for the same duration after being treated with PD98059 (10 μM) for 8 hours or MEK1 (5 μM) for 4 hours. The concentration and duration of DPP treatment were chosen based on preliminary studies examining its effects on cell growth inhibition and induction of apoptosis.

### Western blotting

Proteins in the total cell lysate (40 μg of protein) were separated on 10% SDS-PAGE and electrotransferred to a polyvinylidene difluoride membrane (Immobilon-P membrane; Millipore, Bedford, MA, USA). After the blot was blocked in a solution of 5% skimmed milk, 0.1% Tween 20 and PBS, membrane-bound proteins were probed with primary antibodies against sCLU, pERK1/2 and ERK1/2 (Santa Cruz Biotechnology, Santa Cruz, CA, USA). The membrane was washed and then incubated with horseradish peroxidase-conjugated secondary antibodies for 30 minutes. Antibody-bound protein bands were detected with enhanced chemiluminescence reagents (Amersham Pharmacia Biotech, Piscataway, NJ, USA) and photographed with Kodak X-Omat Blue autoradiography film (Perkin Elmer Life Sciences, Boston, MA, USA).

### Cell growth inhibition by MTT assay

KH OS, Sa OS, U-2 OS, KH OS/sCLU, KH OS/pCDNA3.1, U-2 OS/sCLU-shRNA1/2 and U-2 OS/shRNA cells were seeded at a density of 1 × 10^4^ cells per well in 96-well microtiter culture plates. After overnight incubation, medium was removed and replaced with fresh medium containing different concentrations of DDP (0 to 10 uM/mL) diluted from a 10 mmol/L stock. On completion of 72 hours of incubation, 20 μL of 3-(4,5-dimethylthiazol-2-yl)-2,5-diphenyltetrazolium bromide (MTT) solution (5 mg/mL in PBS) were added to each well and incubated further for 2 hours. Upon termination, the supernatant was aspirated and the MTT formazan formed by metabolically viable cells was dissolved in 100 μL of isopropanol. The plates were mixed for 30 minutes on a gyratory shaker, and absorbance was measured at 570 nm using a plate reader. For the KH OS/sCLU and KH OS/pCDNA3.1 clones, they were treated with PD98059 (10 μM) for 8 hours, then treated with DPP (1 to 10 μM/mL) for 72 hours. U-2 OS/sCLU-shRNA1/2 and U-2 OS/shRNA clones were treated with MEK1 (5 μM) for 8 hours, then treated with DPP (1 to 10 μM/mL) for 72 hours. MTT was used as above to detect cell growth.

### Tumor growth xenograft study

The research was approved by the ethics committee at the affiliated hospital of, Qingdao university.Male BALB/c mice (six weeks) were purchased from Criver.Co, Shanghai, China. They were housed under a standardized light/dark cycle at room temperature of 24 ± 1°C and humidity of 60 ± 10% with food and water *ad libitum*. A 25-μl volume containing 1 × 10^7^ viable cells (U-2 OS, U-2 OS/sCLU-shRNA-1 and U-2 OS/sCLU-shRNA-2, KH OS, KHOS/sCLU, control KHOS/pCDNA3.1 and U-2 OS/shRNA) was subcutaneously inoculated in the upper left flank on day 1. When the diameters of tumors were > 5 mm, the mice in each group were treated with intra-tumoral injection of 8 mg/kg DDP in sterile water accordingly twice a week for 4 weeks. All solutions used sterile water as solvent. Tumor volume was determined with caliper and calculated using (width^2^ × length)/2 twice a week. To avoid unnecessary harm, drugs were injected gently. The conditions of the animals were monitored daily for evidence of illness. Mice showing severe distress including infection, ulceration, cachexia, inability to ambulate and those which were moribund were euthanized in accordance with animal care protocol.

### Statistical analysis

For each protocol, three independent experiments were performed. Results were expressed as the mean ± SEM. Statistical calculations were performed using SPSS 11.0 (SPSS Inc., Chicago, IL, USA). Differences in measured variables among experimental and control groups were assessed using *Χ*^*2*^ test. *P* < 0.05 was considered statistically significant.

## Results

### sCLU expression positively correlates with phosphorylated ERK1/2 (p-ERK1/2)

**Figure 1:**
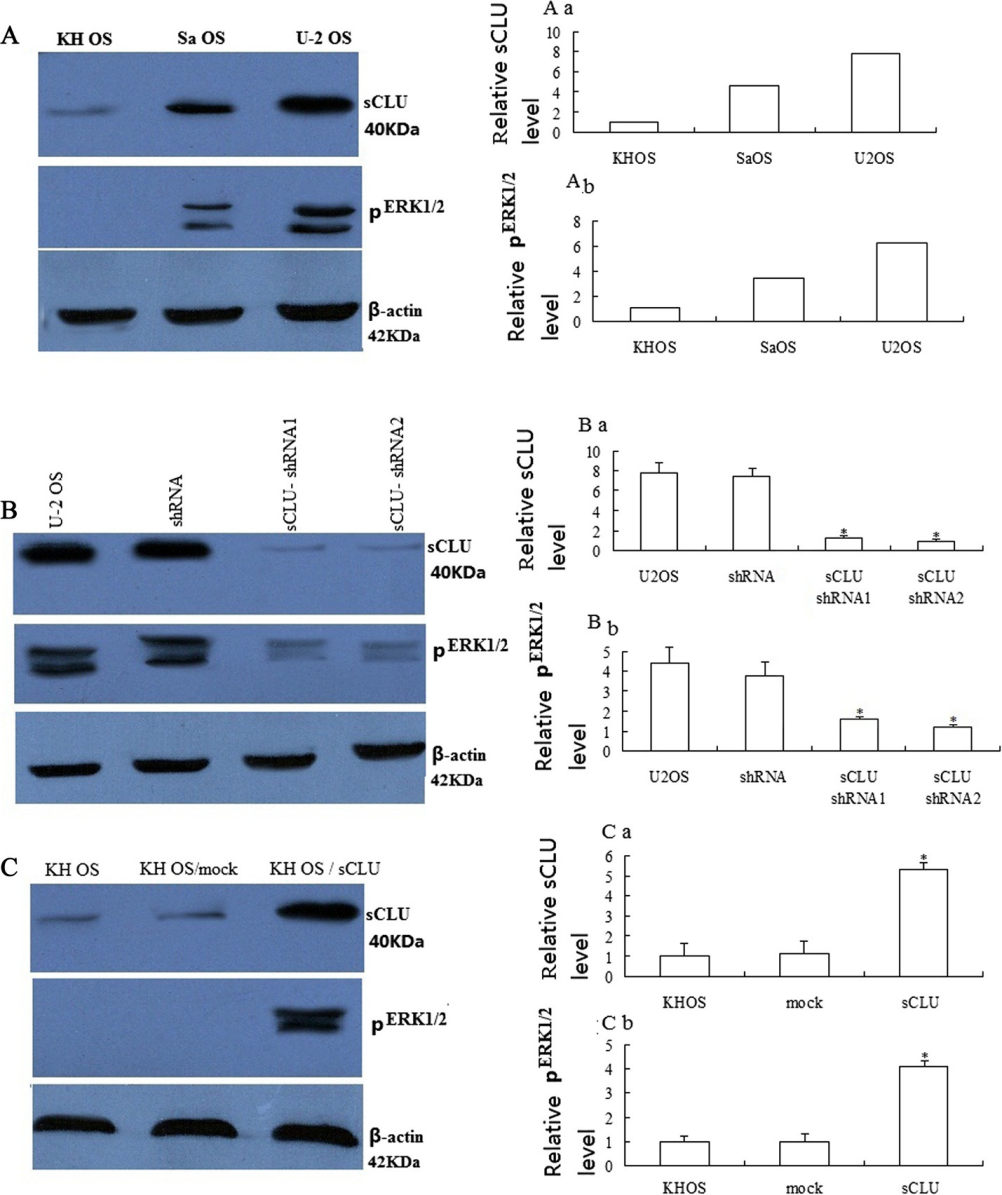
**Clusterin (CLU) expression positively correlates with pERK1/2 expression. (A)** Western blot analysis was performed to determine basal expression of CLU and pERK1/2 expression in indicated OS cell lines. **(B)** Western blot showing 90% reduction in CLU levels in two U-2 OS subclones stably transfected with sCLU-specific shRNA. Untreated and vector alone-transfected U-2 OS cells show high basal levels of CLU expression. **(C)** Western blot showing 95% increase in CLU levels in KH OS/sCLU clones stably transfected with sCLU. Untreated and vector alone-transfected KH OS cells show low basal levels of CLU expression. The membranes were stripped and reprobed with anti-β-actin antibody to ensure even loading of proteins in each lane. Results shown are from representative experiments repeated at least twice with similar findings. Versus control, *P < 0.05.

It has recently reported that aberrant expression of sCLU was shown in OS cells [23, 24], and sCLU up-regulation in the multi-drug resistant OS cells relates to enhanced drug resistance [24]. Furthermore, sCLU knockdown in human OS cells induces significant reduction of cellular growth and higher rates of spontaneous endogenous apoptosis [16]. However, the mechanism by which sCLU affects these functions was not delineated. Previous studies found CLU regulates aggressive behavior and chemosensitivity through modulating ERK1/2 signaling in pancreatic cancer [15], breast cancer [23], and lung cancer cells [22]. We hypothesized that CLU might affect the ERK1/2 pathway in the OS cells. To test this hypothesis, first, we tested three OS cell lines for CLU and pERK1/2 expression. Using Western blot analysis, we observed that KH OS, Sa OS, and U-2 OS express very low, moderate, and high endogenous steady-state CLU amounts, respectively. The cell lines that expressed high basal levels of CLU protein contained high levels of pERK1/2 (Figure 1A).

We further confirmed the positive correlation between CLU and pERK1/2 expression in two stably CLU shRNA-transfected U-2 OS sublines (U-2 OS/sCLU-shRNA-1, U-2 OS/sCLU-shRNA-2) and stably CLU-transfected KH OS sublines (KH OS/sCLU) (Figure 1B). The two U-2 OS clones showed 90% decrease in CLU expression compared with the parental U-2 OS cells (Figure 1B). Importantly, the decrease in CLU expression in both the clones was associated with a parallel decrease in pERK1/2 expression (Figure 1B). The KH OS/sCLU clones showed 95% increase in CLU expression compared with the parental KH OS cells (Figure 1C). The increase in CLU expression in KH OS/sCLU clones was associated with a parallel increase in pERK1/2 expression. This positive correlation between CLU and pERK1/2 expression in OS cell lines suggested that CLU might be involved in the regulation of pERK1/2 expression.

### OS cell lines vary in resistance to DDP

**Figure 2:**
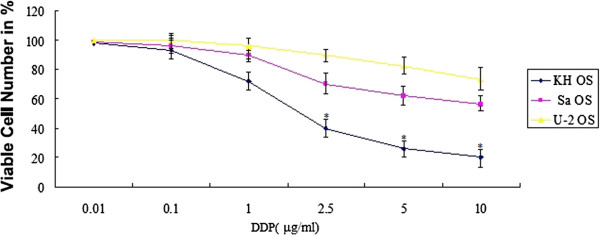
**Osteosarcoma (OS) cells have differing levels of native resistance to cisplatin (DDP).** Human OS lines KH OS, Sa OS, and U-2 OS were treated with increasing concentrations of DDP (0 to 10 μg/mL) for 72 hours. The viabilities indicated on the y axis were determined by 3-(4,5-dimethylthiazol-2-yl)-2,5-diphenyltetrazolium bromide (MTT) assays and normalized to control. Data shown are means ± SE for n = 3 independent experiments. *P < 0.05.

We examined the relative sensitivity of three commonly used OS lines (KH OS, Sa OS, and U-2 OS) to DDP *in vitro*. Cells were treated with different concentrations of DDP (0 to 10 μg/mL) for 72 hours and the number of surviving cells was analyzed (Figure 2). The KH OS cells were sensitized to DDP, and the U-2 OS cells were resistant to DDP treatment. The same sensitivities were obtained when the effects of DDP were analyzed on apoptosis using flow cytometry (FCM) (data not shown). These data supported the suggestion that intrinsic sCLU levels in the OS cells are correlated with sensitivity to DDP.

### DDP treatment induces sCLU up-regulation in the OS cells

**Figure 3:**
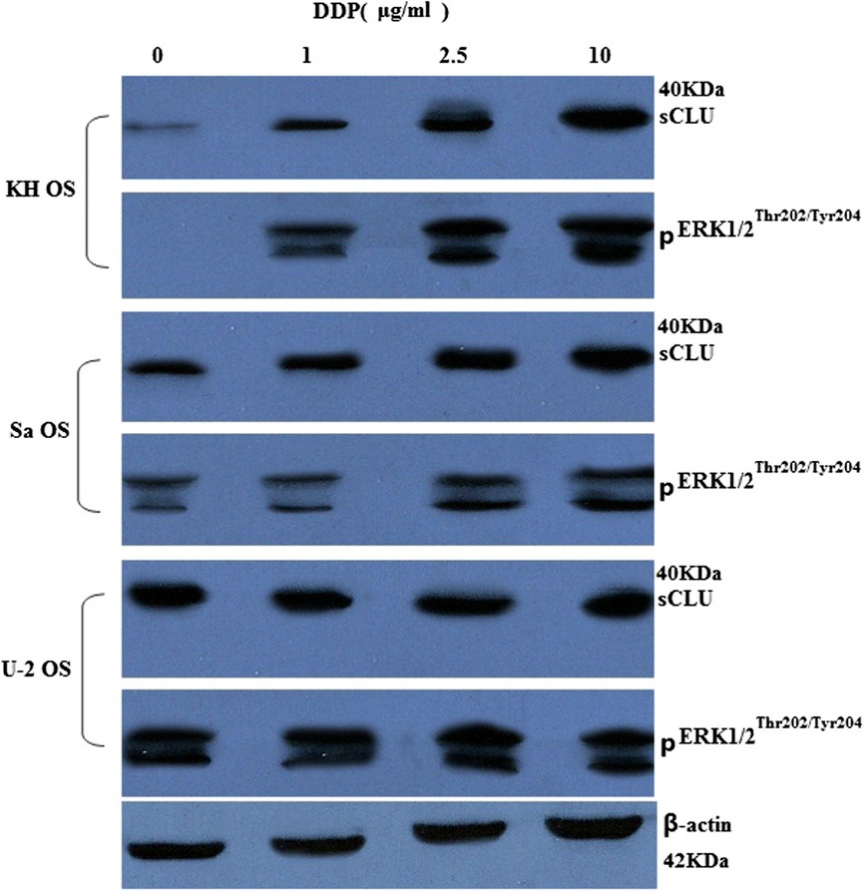
**Cisplatin (DDP) treatment induces sCLU and pERK1/2 up-regulation.** Human OS lines KH OS, Sa OS, and U-2 OS were treated with increasing concentrations of DDP (0 to 10 μg/mL) for 72 hours. Western blot analysis was done to determine expression of clusterin and pERK1/2 in indicated OS cell lines. The membranes were stripped and reprobed with anti-β-actin antibody to ensure even loading of proteins in each lane. Results shown are from representative experiments repeated at least twice with similar findings.

Cells were treated with different concentrations of DDP (0 to 10 μg/mL) for 72 hours. Our studies showed that the protein expression levels revealed a minimal CLU up-regulation in the U-2 OS cells and a significant induction in the KH OS and moderate induction in the Sa OS cells (Figure 3).

### DDP treatment induces sCLU-dependent pERK1/2 up-regulation in the OS cells

Cells were treated with different concentrations of DDP (0 to 10 μg/mL) for 72 hours. The protein expression levels revealed a minimal pERK1/2 up-regulation in the U-2 OS cells and a significant induction in the KH OS and moderate induction in the Sa OS cells (Figure 3). However, when the cells were treated with PD98059 for 8 hours followed by DPP (0 to 10 μg/mL) for 72 hours, expression of pERK 1/2 was clearly suppressed in all cell lines treated for 72 hour with DPP (data not shown).

### sCLU regulates osteosarcoma cell growth by modulating ERK1/2 expression

**Figure 4:**
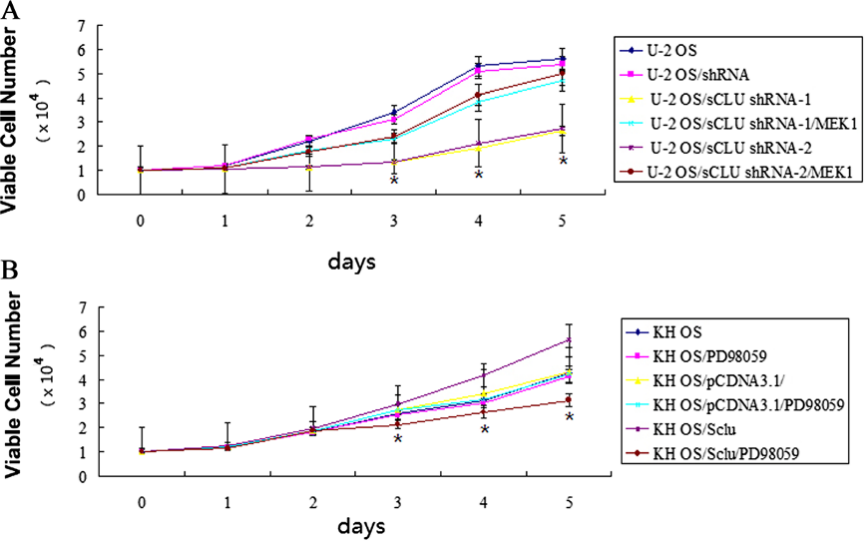
**sCLU regulates osteosarcoma cell growth by modulating ERK1/2 expression. (A)** Cell proliferation was assessed at the indicated times by 3-(4,5-dimethylthiazol-2-yl)-2,5-diphenyltetrazolium bromide (MTT) assays. Data are from three independent experiments. *P < 0.05, compared to the control group. **(B)** Cell proliferation was assessed at the indicated times by MTT assays. Data are from three independent experiments. *P < 0.05, compared to the KH OS/sCLU group.

KH OS and U-2 OS cells were selected for growth assays because they represent two extreme opposite cases as far as the endogenous CLU amount. To determine whether sCLU shRNA had an inhibitory effect on OS cell growth, we first performed determination of U-2 OS cell proliferation with the MTT assay. Figure 4A showed that the growth curves for CLU shRNA-transfected U-2 OS sublines (U-2 OS/sCLU-shRNA-1 and U-2 OS/sCLU-shRNA-2) were significantly lower than those for controls and mock shRNA-transfected U-2 OS sublines for five days of incubation. However, when the U-2 OS/sCLU-shRNA-1 and U-2 OS/sCLU-shRNA-2 cells were treated with MEK1 (5 μM) for 4 hours to activate the ERK1/2, the growth curves were significantly elevated compared to the growth curves in the U-2 OS/sCLU-shRNA-1 and U-2 OS/sCLU-shRNA-2 cells (Figure 4A).

To determine whether sCLU had an increased effect on OS cell growth, we then performed determination of KH OS cell proliferation with the MTT assay. Figure 4B shows that the growth curves for stably CLU-transfected KH OS sublines (KH OS/sCLU) were significantly higher than those for control cells and mock-transfected KH OS sublines for five days of incubation. These results indicated that knockdown of sCLU inhibits cell proliferation, and sCLU overexpression promotes cell proliferation.

To determine whether sCLU regulates OS cell growth by modulating ERK1/2 expression, KH OS/sCLU clones and KH OS were treated with PD98059 for 8 hours to inhibit ERK1/2 activity, we then performed determination of KH OS cell proliferation with MTT assay. Figure 4B showed that the growth curves for PD98059 treated-KH OS/sCLU were decreased compared to the KH OS/sCLU clones for five days of incubation.

### Overexpression of sCLU decreases DPP-induced cytotoxicity in OS cells by ERK1/2 activation

**Figure 5:**
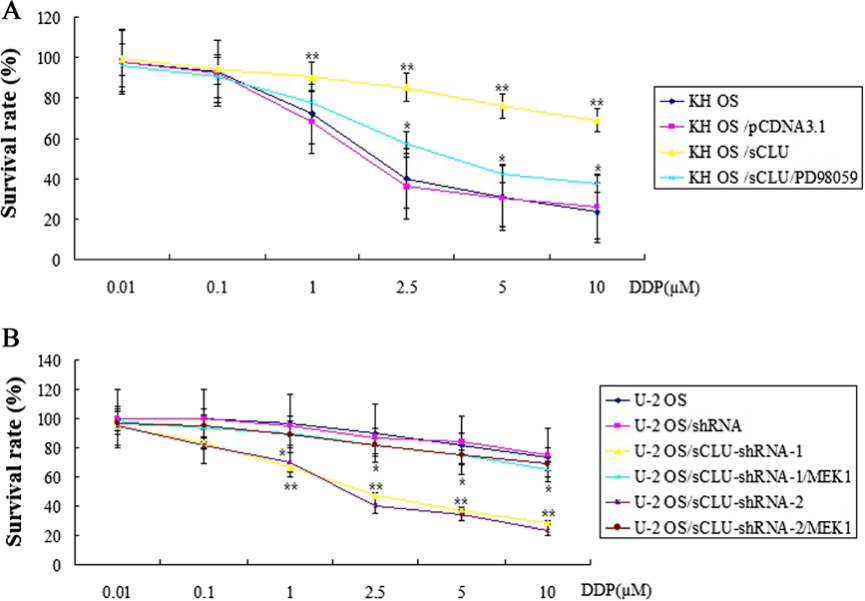
**sCLU regulates the cisplatin (DDP)-induced cytotoxicity in the osteosarcoma (OS) cells. (A)** The KH OS or sCLU stably transfected KH OS cells (KH OS/sCLU) were treated with 0.01 to 10 μM/ mL DPP for 72 hours in the presence or absence of 10 mM PD98059. Cell viability was determined using a 3-(4,5-dimethylthiazol-2-yl)-2,5-diphenyltetrazolium bromide (MTT) assay. The results were obtained from three independent experiments. Versus KHOS/sCLU, *P < 0.01; versus KH OS, **P < 0.01. (*Χ* ^2^ test). **(B)** The sCLU-shRNA stably transfected U-2 OS cells (U-2 OS/sCLU-shRNA-1 and U-2 OS/sCLU-shRNA-2) were treated with 0.01 to 10 μM/mL DPP for 72 hours in the presence or absence of 5 μM MEK1. Cell viability was determined using an MTT assay. The results (mean ± SEM) were obtained from three independent experiments. sCLU shRNA1/2 groups versus MEK1 groups, *P < 0.01; sCLU shRNA1/2 groups versus U-2 OS control groups, **P < 0.01 (*Χ* ^2^ test).

To determine whether the overexpression of CLU by CLU cDNA transfectants decreases DPP-induced cytotoxicity, cell viability assay was performed at 72 hours after DPP treatment (0.01 to 10 μM/mL) using the MTT assay. The sCLU stably transfected KH OS cells (KH OS/sCLU) showed significantly (*P* < 0.01) lower susceptibility to DPP than parental KH OS cells and mock transfected KH OS cells (Figure 5A). However, when the KH OS/sCLU cells were treated with PD98059 (10 μM) for 8 hours to decrease the ERK1/2 activity, the chemosensitivity to DPP in KH OS/sCLU cells was significantly increased (*P* < 0.01) (Figure 5A).

### Silencing of sCLU enhances DPP-induced cytotoxicity in OS cells by ERK1/2 inactivation

To determine whether the inhibition of CLU expression by CLU shRNA transfectants enhances DPP-induced cytotoxicity, cell viability assay was performed at 72 hours after DPP treatment (1 to 10 μM/mL) using the MTT assay. The sCLU-shRNA stably transfected U-2 OS cells (U-2 OS/sCLU-shRNA-1 and U-2 OS/sCLU-shRNA-2) showed significantly (*P* < 0.01) higher susceptibility to DPP than parental U-2 OS cells and mock shRNA-transfected U-2 OS cells (Figure 5B). However, when the U-2 OS/sCLU-shRNA-1 and U-2 OS/sCLU-shRNA-2 cells were treated with MEK1 (5 μM) for 4 hours to activate the ERK1/2, the chemosensitivity to DPP in U-2 OS/sCLU-shRNA-1 and U-2 OS/sCLU-shRNA-2 cells was significantly decreased (*P* < 0.01) (Figure 5B).

### sCLU knockdown attenuates tumor growth and augments DPP activity by ERK1/2 inactivation *in vivo*

Given the remarkable effects of sCLU *in vitro*, we first tested the effect of sCLU overexpression in an orthotopic mouse model of OS (KH OS). To simulate treatment of advanced small-volume disease, therapy was initiated two week after the injection of the tumor cells. When the diameters of tumors were > 5 mm, the mice in each group were then treated with intra-tumoral injection of 8 mg/kg DDP in sterile water twice a week for 4 weeks. A total of 36 mice were divided into 6 groups (n = 6 mice per group): KH OS, KH OS + DDP; KHOS/sCLU, KHOS/sCLU + DDP; KHOS/pCDNA3.1, KHOS/pCDNA3.1 + DDP. After four weeks of therapy, the animals were sacrificed.

**Figure 6:**
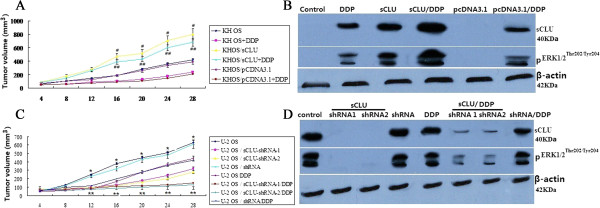
**Response of osteosarcoma (OS) cells and sCLU or shRNA-sCLU transfected OS cells grown as orthotopic xenografts to cisplatin (DDP). (A)** KH OS, KHOS/sCLU, KHOS/pCDNA3.1 tumors were treated with DDP for four weeks. ^#^ *P* < 0.05, KHOS/sCLU versus KHOS (or KHOS/pCDNA3.1); ^##^ *P* < 0.05, KHOS/sCLU/DDP versus KHOS/DDP (or KHOS/pCDNA3.1 DDP); **(B)** sCLU and pERK1/2 expression were detected by Western blot assay. **(C)** U-2 OS, U-2 OS/sCLU-shRNA-1, U-2 OS/sCLU-shRNA-2 and U-2 OS/shRNA tumors were treated with DDP for 4 weeks. *P < 0.05, U-2 OS(or U-2 OS/shRNA) versus U-2 OS/sCLU-shRNA-1 and U-2 OS/sCLU-shRNA-2; **P < 0.05, U-2 OS/sCLU-shRNA-1/DDP and U-2 OS/sCLU-shRNA-2/DDP versus U-2 OS/DDP (or U-2 OS/shRNA/DDP); **(D)** sCLU and pERK1/2 expression were detected by Western blot assay.

Over-expression of sCLU has a dramatic effect on the growth rate of orthotopic xenografts. As shown in Figure 6A, KH OS and KH OS/pCDNA3.1 cells grow slowly. In contrast, KH OS/sCLU cells grow more rapidly, reaching an average volume of 800 mm^3^ by 4 weeks, although the growth rate of individual tumors is quite variable. DDP significantly decreases the volume of the tumors derived from untransfected KH OS cells or mock transfected cells ( KH OS/pCDNA3.1), but has no statistically significant effect on the volume of KH OS/sCLU tumors (Figure 6A). sCLU overexpression was confirmed from Western blot data. pERK1/2 was activated in the KHOS/sCLU groups than the KH OS/pCDNA3.1 and KH OS groups (Figure 6B).

We next tested the effect of sCLU knockdown in an orthotopic mouse model of U-2 OS. A total of 48 mice were divided into 8 groups (n = 6 mice per group): U-2 OS, U-2 OS/sCLU-shRNA-1, U-2 OS/sCLU-shRNA-2 and U-2 OS/shRNA, (U-2 OS, U-2 OS/sCLU-shRNA-1, U-2 OS/sCLU-shRNA-2 and U-2 OS/shRNA) + DDP groups. After four weeks of therapy, the animals were sacrificed. Knockdown of sCLU has a dramatic effect on the growth rate of orthotopic xenografts. As shown in Figure 6C, U-2 OS and U-2 OS/shRNA (control) tumors grow rapidly. In contrast, U-2 OS/sCLU-shRNA-1 and U-2 OS/sCLU-shRNA-2 cells grow more slowly. DDP did not significantly decreases the tumor volume derived from untransfected U-2 OS cells or mock transfected cells (U-2 OS/shRNA), but has statistically significant effect on the volume of U-2 OS/sCLU-shRNA-1 and U-2 OS/sCLU-shRNA-2 tumors (Figure 6C). Knockdown of sCLU was confirmed from Western blot data. pERK1/2 was significantly inhibited in the sCLU shRNA1/ 2 transfected groups compared to the controls (Figure 6D).

## Discussion

Inhibition of sCLU has repeatedly and consistently been shown to sensitize OS cells *in vitro* and *in vivo* to the chemotherapy [17, 18, 24]. The mechanism by which sCLU activation in these cancer cells confers chemoresistance is unclearBecause CLU was first discovered as a survival protein in prostate tumor cells, a push was made to investigate whether androgen ablation, irradiation, and paclitaxel treatment, which are common strategies for treatment of this cancer, upregulated CLU. All of these modalities were found to readily induce sCLU expression in human prostate tumor cells [8, 25]. sCLU was also induced in pancreatic cancer [15], breast cancer [26], cervical cancer [27] and bladder cancer [28] by exposure to various cytotoxic agents, leading to a survival advantage. It was clearly shown that the levels of sCLU in the treated samples were markedly higher than those before treatment, thus suggesting that sCLU expression is an adaptive response to provide cytoprotection against the anticancer regimen.

In terms of its induction, the gene for sCLU has been cloned and analysis of its promoter region has revealed several transcription regulators reported to be involved in gene transcription. Transcription factors that have been shown to interact with the CLU promoter and regulate its function include Egr-1 [29], Heat Shock Factor 1/2 [30] and c-MYC [31]. CLU could regulate TGF-beta signaling pathway by modulating the stability of Smad2/3 proteins [32]. Another pathway by which sCLU might act is via NF-κB. Being a protein stabilizer, sCLU apparently can also stabilize IKBα, thus preventing its degradation which is needed to release the p50/p65 NF-κB heterodimer for entry into the nucleus to act as a transcription factor [33]. Another means by which sCLU can affect cell survival is via its receptor. Interestingly, sCLU has been shown to bind its cell surface receptor, megalin, in a rat prostate cell line and induce AKT activation which then can phosphorylate Bad, causing a decrease in cytochrome c release, thus favoring cell survival [34]. Recent studies have demonstrated that sCLU could regulate ERK1/2 activity in renal cell cancer [20], pancreatic cancer [15], monocytes/macrophages [21] and lung cancer [22].

In this study, we demonstrated that overexpression of sCLU was resistant to DDP both *in vitro* and *in vivo* in KH OS cells, and knockdown of sCLU sensitized U-2 OS cells to DDP both *in vitro* and *in vivo*. Furthermore, sCLU is readily induced by the therapeutic agent DDP *in vitro* and *in vivo*. So, sCLU targeting as a means to chemosensitize clinically established drugs could be a potent strategy to overcome drug resistance.

Although sCLU confers DDP resistance in OS cells, the signaling pathway was unclear. ERK activation has been identified as a potential survival pathway in cancers, including OS, and pERK1/2 plays a critical role in drug resistance [16, 19, 35, 36]. It has shown previously that sCLU plays an important role in regulating ERK1/2 signaling [15, 20–22]. We next studied whether sCLU regulates the chemosensitivity to DPP by modulating ERK1/2 in OS cells. Our results have shown that sCLU silencing by sCLU-shRNA transfection sensitizes U-2 OS cells to DDP treatment *in vitro* and *in vivo*, followed by inhibition of pERK1/2 activation. Conversely, transfection with a pCDNA3.1-sCLU vector to KH OS cells promotes DDP resistance *in vitro* and *in vivo*, followed by pERK1/2 activation. These data demonstrate that sCLU regulates chemosensitivity to DPP via a pERK1/2 dependent signaling pathway.

## Conclusions

In summary, sCLU plays a major role on the effects of DDP, protecting OS cells from the effects of DDP. Because sCLU confers survival advantage to OS cells and is readily induced by DDP, sCLU targeting as a means of chemosensitizing clinically established drugs could be a potent strategy to overcome drug resistance.

## Abbreviations

CLU: Clusterin
ERK1/2: Extracellular signal-regulated protein kinases 1 and 2
DMEM: Dulbecco's modification of Eagle's medium Dulbecco.
